# Fabrication of Surface Plasmon Resonance Sensor Surface with Control of the Non-Specific Adsorption and Affinity for the Detection of 2,4,6-Trinitrotoluene Using an Antifouling Copolymer

**DOI:** 10.3389/fbioe.2014.00010

**Published:** 2014-04-29

**Authors:** Rui Yatabe, Takeshi Onodera, Kiyoshi Toko

**Affiliations:** ^1^Research and Development Center for Taste and Odor Sensing, Kyushu University, Fukuoka, Japan; ^2^Graduate School of Information Science and Electrical Engineering, Kyushu University, Fukuoka, Japan

**Keywords:** trinitrotoluene, surface plasmon resonance, immunosensor, surface-initiated atom transfer radical polymerization, non-specific adsorption, self-assembled monolayer, displacement assay

## Abstract

We fabricated a surface plasmon resonance (SPR) sensor using a hydrophilic polymer for the highly sensitive detection of 2,4,6-trinitrotoluene (TNT). The hydrophilic polymer was made from mono-2-(methacryloyloxy)ethylsuccinate (MES) and 2-hydroxyethylmethacrylate (HEMA) by surface-initiated atom transfer radical polymerization. The detection of TNT was carried out by displacement assay with the SPR measurement. In displacement assay, the affinity between anti-TNT antibody and the sensor surface, affects to the sensitivity. In the SPR measurement, non-specific adsorption should be controlled because SPR sensor cannot discriminate between specific and non-specific adsorption. Therefore, the affinity and non-specific adsorption were controlled by changing the ratio of HEMA to MES. A detection limit of 0.4 ng/ml (ppb) for TNT was achieved using a sensor surface with the lowest affinity without non-specific adsorption.

## Introduction

Since the terrorist attacks in the USA on September 11, 2001, the threat to the world has shifted to terrorism from war between nations. The threat of explosives has spread to our living space from the battlefield. Explosives are used by terrorists at places where people gather, such as airports and shopping malls. In airports, sniffer dogs are employed for the detection of explosives. However, if a sensor were capable of measurement with high sensitivity for explosive compounds, it would be possible to uncover bombs more effectively.

The detection of explosives has been attempted by various other methods. In one method, the detection of 2,4,6-trinitrotoluene (TNT) or 1,3,5-trinitroperhydro-1,3,5-triazine (research department explosive, RDX) was performed using a laser (Roberson and Sausa, 2010). This method has the advantage that real-time measurement is possible. Similar to this method, there is a method of using an optical fiber in its optical system (Bakaltcheva et al., 1999; Van Bergen et al., 2000). In an electrochemical method, the chemical adsorption between SiO_2_ nanostructures and nitroaromatic compounds is employed (Zhang et al., 2006). This method is simple and suitable for portable sensors. For another method, a localized surface plasmon resonance (L-SPR) sensor was developed using an imprinted nanostructure of gold particles (Riskin et al., 2009). The advantage of this method is ultrahigh sensitivity to TNT. In another similar method, detection was carried out using the quenching of fluorescence by nitroaromatic compounds (Yang and Swager, 1998; Chang et al., 2004). This method has the advantage that the detection can be performed in air. For another method using fluorescence, fluorescence resonance energy transfer (FRET) and DNA are employed (Medintz et al., 2005). The advantage of this method is capable of adapting dynamic range because the structure of sensor surface has high flexibility. In conclusion, much effort has been expended on the detection of explosives.

We previously realized an ultrahigh-sensitivity TNT sensor using a surface plasmon resonance (SPR) sensor and an antigen–antibody interaction (Matsumoto et al., 2005; Singh et al., 2007; Mizuta et al., 2008; Onodera, 2013). This SPR sensor has high sensitivity to changes in the refractive index upon the adsorption of substances on a thin gold surface. The antigen–antibody interaction, which is an immunoreaction, has high selectivity to a target compound. The TNT sensor, which has high selectivity and sensitivity, operates under combination of these principles.

It is advantageous to arrange the binding sites of the TNT antibody in three dimensions for the detection of TNT at low concentrations. We realized the sensor surface by a method in which a dendrimer or a polymer is immobilized on the surface, or a method in which the polymer chain grows from the surface (Mizuta et al., 2010; Yatabe et al., 2013a,b).

We have used surface-initiated atom transfer radical polymerization (SI-ATRP) to fabricate a polymer surface (Zhao and Brittain, 2000). This fabrication has several steps. First of all, an initiator compound for ATRP is immobilized on the surface as a self-assembled monolayer (SAM). Next, the reaction solution, containing the monomer, catalyst, and reducing agent, is brought into contact with the surface. Then, the polymer chain grows from the initiator on the surface. In ATRP, the polymer chain grows while alternately repeating propagation and dormant states (Matyjaszewski and Xia, 2001). In the propagation state, the growth point has a radical and reacts with a monomer. If the proportion of the propagation state is higher, the polymerization reaction progresses faster, but it often terminates because radicals are presenting high concentrations. Thus, it is difficult to control the polymerization. However, when the reaction conditions are appropriately selected, the proportion of the dormant state becomes much higher than that of the propagation state. As a result, the polymerization seldom terminates because of the low concentration of radicals. This means that it is easy to control the length of the polymer chain by adjusting the reaction time. In addition, another polymerization can restart upon the contact of another reaction solution with the surface after the first polymerization. Therefore, various types of polymer can be connected directly to each other. In conclusion, various types of polymer surface can be easily fabricated by SI-ATRP.

Our sensor detects TNT using the specific binding of an anti-TNT antibody to the sensor surface by an antigen–antibody interaction. Thus, it is difficult to detect TNT when non-specific adsorption occurs on the surface because the SPR sensor cannot discriminate between specific and non-specific adsorption. It is necessary to fabricate a sensor surface with low non-specific adsorption to realize high sensitivity. There are two main mechanisms for non-specific adsorption. One is electrostatic interaction and the other is hydrophobic interaction. The sensor surface must be electrically neutral and hydrophilic to prevent these interactions. In our previous study, we fabricated a sensor surface with low non-specific adsorption using a copolymer of mono-2-(methacryloyloxy)ethylsuccinate (MES) and diethylaminoethylmethacrylate (DEAEM) (Yatabe et al., 2013b). MES has a carboxyl group on the side chain and is negatively charged. DEAEM has a tertiary amino group on the side chain and is positively charged. In the fabrication method, parts of the carboxyl groups of MES react with the amino group of the TNT analog. The negative charge of unreacted carboxyl groups of MES is neutralized by the positive charge of DEAEM. As a result, the surface is electrically neutral and hydrophilic. In addition, a high dissociation rate constant, *k*_d_, between the surface and the antibody has been found to be advantageous for highly sensitive detection (Yasuura et al., 2011; Yatabe et al., 2013a). Hence, it is possible to fabricate a sensor surface with a high *k*_d_ by decreasing the concentration of TNT analog on the polymer. If the concentration of the TNT analog is decreased using MES and DEAEM, it is necessary to decrease the amount of MES because the analog is immobilized on the carboxyl group of MES. However, if the ratio of MES to DEAEM is changed, the surface is not electrically neutral and can be affected by non-specific adsorption. Therefore, to prevent this problem, using MES and 2-hydroxyethylmethacrylate (HEMA), which is electrically neutral and hydrophilic, we attempted to fabricate a poly-MES-*co*-HEMA surface. This surface is negatively charged because the carboxyl group of MES is not neutralized. However, when the amount of HEMA is higher than that of MES, it is possible to control the non-specific adsorption because the negative charge becomes weaker. Thus, using sensor surfaces fabricated with various ratios of MES to HEMA, we evaluated the characteristics of non-specific adsorption on the surfaces. In addition, the limit of detection (LOD) of TNT was measured on these surfaces by displacement assay. If it were possible to control *k*_d_, it would be advantageous for displacement assay because the sensor response in the dissociation is used for detection in the assay. To achieve high sensitivity, the non-specific adsorption was controlled for SPR measurement, and the affinity between the antibody and the sensor surface was controlled for displacement assay.

## Materials and Methods

### Materials

Tris(2-pyridylmethyl)amine (TPMA, Sigma Aldrich, MO, USA), copper chloride (CuCl_2_, Nacalai Tesque, Inc., Japan), and ascorbic acid (Wako Pure Chemical Industries, Japan) were used in the ATRP reaction. MES and HEMA were obtained as monomers from Sigma Aldrich (MO, USA). Bis[2-(2′-bromoisobutyryloxy)undecyl]disulfide (DTBU) from Sigma Aldrich (MO, USA) was prepared to form an SAM as an initiator for ATRP. *N*-hydroxysuccinimide (NHS) and 1-ethyl-3-(3′-dimethylaminopropyl) carbodiimide hydrochloride (EDC) were purchased as an amine coupling kit from GE Healthcare Bio-sciences (Uppsala, Sweden). 2,4-Dinitrophenyl-e-aminocaproyl-NHNH2 (DNP-Hdrz) was prepared as a TNT analog from Biosearch Technologies, Inc. (USA). Mouse anti-TNT monoclonal antibody (anti-TNT antibody) was obtained from Strategic Biosolutions (DE, USA). A TNT aqueous solution with a concentration of 21.8 ppm was purchased from Chugoku Kayaku (Hiroshima, Japan). Bovine serum albumin (BSA, Wako Pure Chemical Industry, Japan) and lysozyme (Sigma Aldrich, MO, USA) were used to evaluate non-specific adsorption. All other chemicals were purchased either from Tokyo Chemical Industry (Japan), Wako Pure Chemical Industries, Inc. (Japan), or Kanto Chemical, Co. (Japan). All aqueous solutions were prepared with Milli-Q water obtained from a Milli-Q system (Millipore, MA, USA).

### Fabrication of sensor chip surface

**Figure 1:**
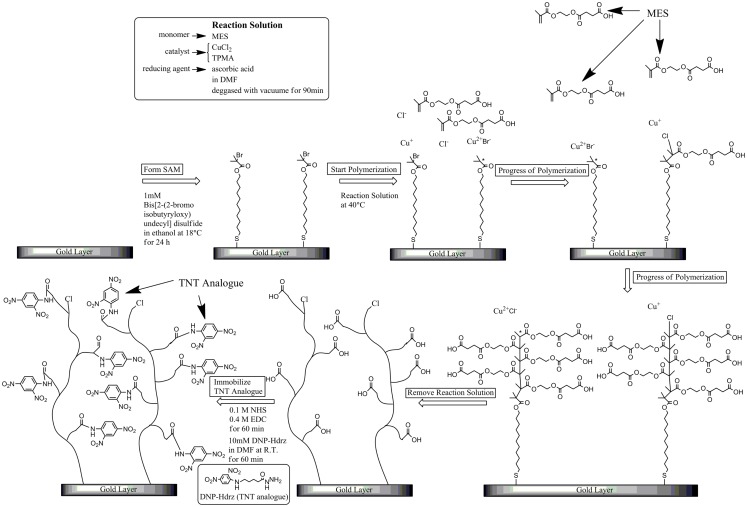
**Fabrication procedure of polymer-based sensor surface by SI-(AGET)ATRP**.

SIA Kit Au (GE Healthcare Bioscience), which contains sensor chips with an unmodified gold layer of ca. 50 nm thickness, was used for the immobilization of various reagents on the surface. Figure 1 shows the fabrication procedure of a sensor surface using SI-ATRP. This figure shows the case in which the monomers were not mixed to simplify the explanation. First, the sensor chip was cleaned in a mixed solution of Milli-Q water, ammonia solution, and hydrogen peroxide with a 5:1:1 volume ratio at 90°C for 20 min. After that, the sensor chip was immersed in 1 mM DTBU (in ethanol) for 24 h at 18°C to form an SAM with an initiator for ATRP. After the sensor chip was cleaned in ethanol by ultrasonic cleaning, it was immersed in a reaction solution at 40°C to induce activator-generated electron transfer for atom transfer radical polymerization (AGET–ATRP). The reaction solution was prepared by mixing a monomer, a catalyst solution, and a reducing agent. The catalyst solution was a mixed solution of 1 mM CuCl_2_ and 10 mM TPMA in *N,N*-dimethylformamide (DMF). The reducing agent was a 1 mM ascorbic acid solution in DMF. These solutions were degassed in vacuum for 1.5 h, and mixed immediately before the polymerization. The reaction solution contained the monomer, CuCl_2_, TPMA, and ascorbic acid. After the polymerization, the reaction solution was removed using DMF. The thickness of the polymer layer in air was measured using a SpecEl-2000-VIS spectroscopic ellipsometer (Mikropack GmbH, Germany). Table 1 shows the reaction conditions and the thickness of the polymer layer under each set of conditions. These reaction conditions were adjusted to obtain polymer layers with about 10 nm thickness. Then, the sensor chip was immersed in a mixed solution of 0.4 M EDC (in water) and 0.1 M NHS (in DMF) at a 1:1 volume ratio for 1 h to activate the carboxyl groups of the polymer as NHS esters. Next, the chip was immersed in 10 mM DNP-Hdrz (in DMF) for 1 h to combine the amino group of the DNP-Hdrz and the activated carboxyl group of the polymer. Then, the chip was immersed in 0.5 wt.% sodium dodecyl sulfate for cleaning. Finally, it was rinsed in Milli-Q water to complete the fabrication of the sensor chip with binding sites of an anti-TNT antibody on the polymer.

**Table 1:** **The reaction conditions and the thickness of polymer layer; diluted by DMF: monomer solution in ATRP reaction solution was diluted by DMF with some fold volume; reaction time: for example, “600 × 3” means that the polymerization thrice was performed for 600 s**.

Sample	Monomer		ATRP reaction solution					Reaction time (s)	Polymer thickness (nm)
	Molar ratio		Molar ratio				Diluted by DMF	
	MES	HEMA	Monomer	TPMA	CuCl_2_	Ascorbic acid	
A	1	0	100000	10	1	1	–	600 × 3	11.8
B	1	1	100000	10	1	1	–	600 × 3	11.6
C	1	2	100000	10	1	1	–	600 × 1	8.6
D	1	5	100000	10	1	1	–	600 × 2	8.8
E	1	10	20000	10	1	0.5	×5	600 × 1	8.3
F	1	100	16667	10	1	2.5	×6	600 × 3	8.1
G	1	1000	20000	10	1	0.5	×5	1200 × 3	8.8

*The reaction solution was newly prepared after each polymerization was completed*.

### Instrument and conditions for SPR

Biacore J (GE Healthcare Bio-sciences) was used for the SPR measurement. HBS-T (10 mM 2-[4-(2-hydroxyethyl)-1-piperazinyl]-ethane sulfonic acid, 150 mM NaCl, 0.005% Tween 20: pH 7.4) was used as a running buffer solution. The measurements were conducted at a constant temperature of 25°C in nitrogen. The flow rate of the solution was 10 μl/min. When the anti-TNT antibody solution was allowed to flow over the DNP-Hdrz immobilized on the chip surface, the antibody was bound to DNP-Hdrz. The SPR sensor can measure the change in the refractive index. The SPR response, which is defined in resonance units (RU), increased with increasing amount of antibody bound on the surface. A resonance angle shift of 0.0001° is defined as 1 RU and is equivalent to a change in mass of 1 pg/mm^2^ on the surface (Stenberg et al., 1991). In most of the experiments, the dissociation of surface-bound antibodies for regeneration was completed when 5 M guanidine hydrochloride (in water) was allowed to flow over the surface. The association rate constant (*k*_a_) and dissociation rate constant (*k*_d_) were calculated from the sensor response curve by BIAevaluation software (version 3.2; GE Healthcare Bio-sciences). The analysis model was 1:1 (Langmuir) binding. To induce association, the antibody at a concentration of 25 ppm was allowed to flow for 5 min. The dissociation was conducted for 5 min.

### TNT detection by displacement assay

In our previous study, the detection of TNT was conducted by inhibition assay. The inhibition assay can realize high sensitivity but requires about 30 min for the measurement. On the other hand, the displacement assay has lower sensitivity than the inhibition assay but is capable of rapid measurement. In this study, TNT detection was carried out by displacement assay (Rabbany et al., 2000; Onodera et al., 2011). There were three steps in the measurement by displacement assay. First of all, when an antibody solution (25 ppm in HBS-T) of known concentration was allowed to flow on the sensor surface for 2 min, the anti-TNT antibodies were bound to TNT analogs (DNP-Hdrz) on the surface by specific adsorption. Next, the running buffer (HBS-T) was automatically allowed to flow on the surface for 40 s. During this time, the antibodies spontaneously dissociated from the surface at a low rate. This step was necessary for the preparation of the next step. At the end of this step, the sensor response was recorded as Δθ_pre_. Next, a TNT solution (in HBS-T) was allowed to flow on the surface for 2 min. At the end of this step, the sensor response was recorded as Δθ_post_. At each concentration of TNT, τ_i_ = Δθ_post_/Δθ_pre_ was calculated [*i* = 0, 1, 2, 3, 4, 5, 6, 8; TNT concentration = 0, 1, 5, 10, 25, 50, 100, 1000 ng/ml (ppb)]. In this study, the displacement ratio was defined by the following formula. The measurement at a concentration of 1000 ng/ml was carried out when the LOD was higher than 100 ng/ml.

(1) $$\text{Displacement ratio}=(\tau_{0}-\tau_{i})/\tau_{0}\;i=1,\;2,\;3,\;4,\;5,\;6,\;7,\;8$$

The dissociation rate increased with the concentration of TNT. The antibodies dissociate from the surface while repeatedly associating and dissociating with the TNT analogs on the surface. When TNT solution is allowed to flow on the surface, the antibodies bind to TNT molecules. The antibodies bound to TNT molecules seldom bind with TNT analogs on the surface. As a result, the dissociation rate increases, when TNT molecules exist on the surface.

## Results and Discussion

### Evaluation of non-specific adsorption on poly-MES-*co*-HEMA based surface

Our sensor detects TNT on the basis of the specific binding between the antibody and antigen (analog) by an immunoreaction. The dependence on the TNT concentration becomes weaker when non-specific adsorption occurs. As a result, it is difficult to detect TNT at low concentrations. Non-specific adsorption can occur on the poly-MES-*co*-HEMA surface because the surface has a slightly negative charge because of MES. Hence, it is necessary to evaluate non-specific adsorption with various ratios of MES to HEMA.

**Figure 2:**
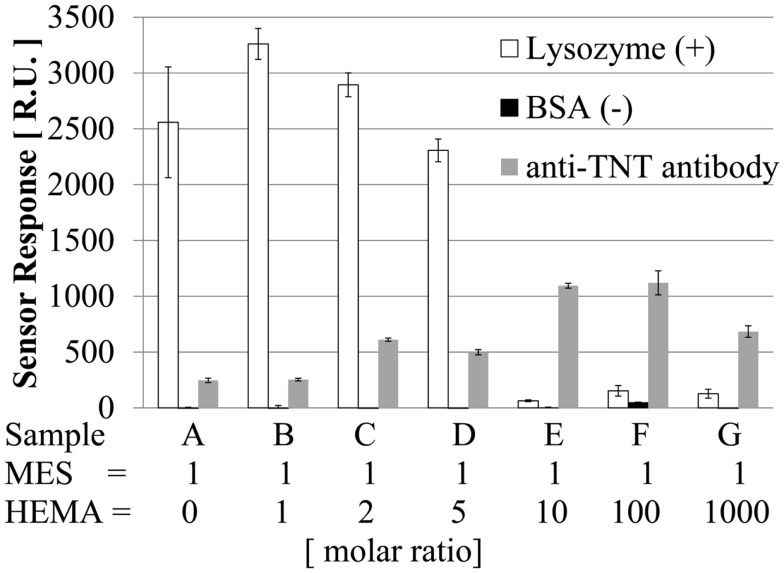
**Adsorption of lysozyme, BSA, and anti-TNT antibody**.

The amount of adsorption on the surface was measured by SPR when 25 ppm anti-TNT antibody, 1000 ppm lysozyme, or 1000 ppm BSA was allowed to flow on the surface for 2 min. The antibody can adsorb on the surface by specific/non-specific adsorption. Lysozyme, which is positively charged, can adsorb on the surface by electrostatic interaction if the surface is negatively charged. BSA, which is negatively charged, can adsorb on the surface if the surface is positively charged. In addition, lysozyme and BSA can adsorb on the surface by a hydrophobic interaction if the surface has hydrophobicity. Figure 2 shows the results of SPR measurement of the surface with various ratios of MES to HEMA. First, the surface was not positively charged, and did not have hydrophobicity because BSA was not adsorbed on the surface under any of the conditions. Second, the amount of lysozyme adsorbed decreased with increasing amount of HEMA. When the amount of HEMA was high, negligible amount of lysozyme were able to adsorb on the surface by electrostatic interaction because the negative charge on the surface became weaker. A surface with low non-specific adsorption was obtained at MES:HEMA ratios of 1:10, 1:100, and 1:1000. Third, at MES:HEMA ratios of 1:0, 1:1, 1:2, 1:5, and 1:10, the amount of antibody adsorbed increased with the amount of HEMA. It is suggested that the antibody we used was negatively charged because of this result and that in our previous study: the amount of antibody adsorbed was large when the polymer surface was positively charged (Yatabe et al., 2013b). Adsorption of the antibody, which can be negatively charged, on the surface was negligible because the surface was negatively charged when the amount of HEMA was small. Next, the amount of antibody absorbed became maximum when the MES:HEMA ratio was 1:10 or 1:100. The amount of antibody absorbed was smaller for the MES:HEMA ratio of 1:1000 than for the ratio of 1:100. For the MES:HEMA ratio of 1:1000, the amount of MES was the smallest among all of the conditions. The binding site of the antibody was DNP-Hdrz, which was immobilized on the carboxyl group of MES. The number of binding sites decreased when the amount of MES was small. It was difficult for the antibody to adsorb on the surface because the amount of immobilized DNP-Hdrz was small. In conclusion, a sensor surface with low non-specific adsorption and capable of immunoreaction was fabricated by mixing MES and HEMA.

### Detection of TNT by displacement assay with surfaces of various affinities

The detection of TNT was performed by displacement assay. The measurement was carried out three times for each concentration of TNT. Calibration curves for the TNT concentration were created by the measurement of each sample. The LOD was calculated by considering three standard deviations at a 0 ppb concentration of TNT (the same as for HBS-T). In addition, the association rate constant *k*_a_ and dissociation rate constant *k*_d_ were calculated using a sensorgram when the running buffer solution was allowed to flow for 5 min after the antibody solution (25 ppm) flowed for 5 min on the surface. Table 2 shows the results. First, the LOD became small with decreasing amount of MES. The *k*_d_ tended to become high with decreasing amount of MES. The amount of antibody adsorbed was small when the ratio of MES was small, as described in Section “Evaluation of Nonspecific Adsorption on Poly-MES-*co*-HEMA Based Surface,” because of the high *k*_d_. A low LOD was realized with a high *k*_d_. The lowest LOD was 0.4 ppb. The measuring time was 5 min.

**Table 2:** Kinetics parameters and the limit of detection (LOD); the LOD was calculated by considering three standard deviations at a 0 ppb concentration of TNT; sensor response *1: it was measured when the injection of TNT solution was started.

Sample	Monomer		Sensor response *1 (R.U.)	Association rate constant *k*_a_ (M s^−1^)	Dissociation rate constant *k*_d_ (s^−1^)	LOD [ng/ml] (ppb)
	Molar ratio	
	MES	HEMA	
A	1	0	201.0	8.43E+04	3.49E−04	271.3
B	1	1	356.3	1.26E+05	2.08E−04	77.1
C	1	2	566.0	1.80E+05	9.16E−04	245.8
D	1	5	431.0	5.04E+04	1.19E−03	37.0
E	1	10	714.1	1.77E+05	1.92E−03	8.4
F	1	100	529.8	1.37E+05	2.65E−03	12.9
G	1	1000	113.0	1.86E+05	2.02E−03	0.4

*Time has elapsed 40 s from when the injection of antibody solution is completed*.

**Figure 3:**
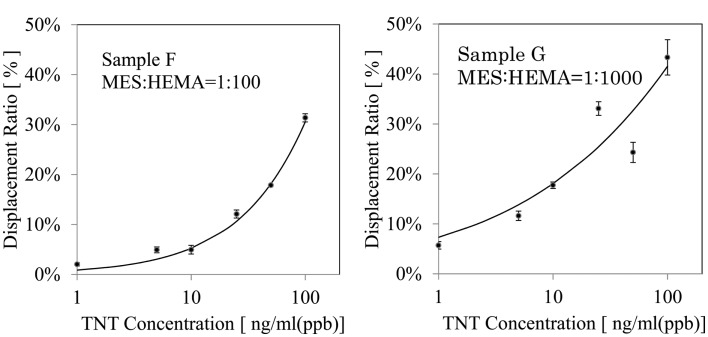
**Response characteristic to TNT: calibration curves obtained by displacement assay using 25 mg/mL (25 ppm) anti-TNT antibody**. The error bar shows the SD of the data.

Next, Figure 3 shows the calibration curves for MES: HEMA = 1:100 and 1:1000. The points on the graph are real data and the lines on the graph are fitted curves. The error bar on each point shows the standard deviation. Table 3 shows the standard deviation of each TNT concentrations. The displacement ratio depended on the concentration of TNT for all data at MES:HEMA = 1:100. The standard deviation data had no tendency. On the other hand, the displacement ratio was independent of the TNT concentrations of 25 and 50 ng/ml (ppb) at MES:HEMA = 1:1000 as indicated by the random distribution of the data points. The standard deviation data tended to increase with the concentration of TNT. The value recorded for the 100 ng/ml sample was not reliable because of the relatively high standard deviation. Therefore, the displacement ratio only depended on the TNT concentration of 1, 5, and 10 ng/ml (ppb) at MES:HEMA = 1:1000. The data “sensor response *1” at MES:HEMA = 1:1000 was the lowest among all the conditions. This means that the amount of antibody adsorbed on the surface in the case of MES:HEMA = 1:1000 was negligible because of high *k*_d_. The response was saturated because most of the antibody had been dissociated from the surface by the high concentration of TNT. Thus, the sensor surface with MES:HEMA = 1:1000 has high sensitivity but cannot measure the concentration of TNT over a wide range. In the detection of explosives, it is more important to be able to discriminate whether or not explosives exist than to measure the concentration of the explosive compounds. Therefore, it is not necessary to be able to measure over a wide range. However, the sensor should have a sufficient measurable range to ensure the reliability of sensing in practical usage. The measurable range and the LOD can be varied by changing the MES:HEMA ratio in our sensor. Hence, the parameters can be optimized by field tests. In conclusion, the sensor surface with a small amount of MES had high sensitivity and a narrow measurable range, and the measurable range can be optimized by changing the MES:HEMA ratio if the range is required to be wider.

**Table 3:** **Standard deviation data of the displacement ratio at MES:HEMA = 1:100 and 1:1000 with each TNT concentrations (*n* = 3)**.

Displacement ratio: standard deviation (%)						
MES:HEMA =	TNT concentration [ng/ml (ppb)]					
	1	5	10	25	50	100
1:100	0.6	1.2	1.7	1.6	0.3	1.6
1:1000	1.5	1.9	1.3	2.7	4.0	7.1

In addition, the sensor surface with poly-MES-*co*-HEMA has another advantage for optimization. Analogs that have amino groups can be used on the poly-MES-*co*-HEMA surface because analogs are immobilized on the carboxyl group of MES by the amine coupling reaction. The advantage is that various analogs can be used; for example, analogs with a carboxyl group can be used if aminoethylmethacrylate (AEMA), which has an amino group, is used in place of MES. It is important to select an appropriate analog because the analog affects the affinity and the characterization of the sensor. In conclusion, it is possible to enhance the capability of the sensor by selecting the analog considering the use of not only MES but also AEMA.

## Conclusion

We fabricated an SPR sensor surface for the detection of TNT by SI-ATRP. The surface was prepared using poly-MES-*co*-HEMA, which was made by mixing the monomers MES and HEMA. MES is negatively charged and can immobilize a TNT analog. HEMA is electrically neutral and hydrophilic. Non-specific adsorption on the surface was controlled by increasing the amount of HEMA. In addition, the LOD and the measurable range could be changed by adjusting the ratio of HEMA to MES. The lowest LOD obtained was 0.4 ppb. We expect that it will be possible to enhance the capability of the sensor by optimizing the ratio of MES to HEMA and selecting an appropriate TNT analog.

## Conflict of Interest Statement

The authors declare that the research was conducted in the absence of any commercial or financial relationships that could be construed as a potential conflict of interest.
